# Associations Between Maternal Antenatal Corticosteroid Treatment and Psychological Developmental and Neurosensory Disorders in Children

**DOI:** 10.1001/jamanetworkopen.2022.28518

**Published:** 2022-08-24

**Authors:** Katri Räikkönen, Mika Gissler, Terhi Tapiainen, Eero Kajantie

**Affiliations:** 1 Department of Psychology and Logopedics, Faculty of Medicine, University of Helsinki, Helsinki, Finland; 2 Department of Knowledge Brokers, Finnish Institute for Health and Welfare THL (Terveyden ja hyvinvoinnin laitos), Helsinki, Finland; 3 Research Centre for Child Psychiatry, University of Turku, Turku, Finland; 4 Department of Molecular Medicine and Surgery, Karolinska Institutet, Stockholm, Sweden; 5 Academic Primary Health Care Centre, Region Stockholm, Stockholm, Sweden; 6 Department of Pediatrics and Adolescent Medicine, Oulu University Hospital, Oulu, Finland; 7 Unit of Clinical Medicine, Medical Research Center Oulu, Oulu University Hospital and University of Oulu, Oulu, Finland; 8 Department of Public Health Solutions, Finnish Institute for Health and Welfare THL (Terveyden ja hyvinvoinnin laitos), Helsinki and Oulu, Finland; 9 Department of Clinical and Molecular Medicine, Norwegian University of Science and Technology, Trondheim, Norway; 10 Children’s Hospital, Helsinki University Hospital and University of Helsinki, Helsinki, Finland

## Abstract

**Importance:**

Corticosteroids administered to women at risk of imminent preterm birth is one of the most effective ways to improve the prognosis of infants born preterm. Scant data about long-term neurodevelopmental and neurosensory outcomes among the treatment-exposed children are mixed, suggesting that not all domains of neurodevelopmental and neurosensory function may be equally affected. Moreover, the long-term outcomes may vary according to whether the treatment-exposed children are being born preterm (<37 weeks and 0 days) or term (≥37 weeks and 0 days).

**Objectives:**

To study whether antenatal corticosteroid treatment is associated with psychological developmental and neurosensory disorders in children born term and preterm and whether the associations persist in a sibling-comparison design.

**Design, Setting, and Participants:**

This population-based retrospective register-linkage study comprised all singleton live births in Finland between January 1, 2006, and December 31, 2017, followed up until December 31, 2018, as well as a sibling comparison among term sibling pairs. Data were analyzed from March 21, 2021, to July 7, 2022.

**Exposures:**

Antenatal corticosteroid treatment.

**Main Outcomes and Measures:**

Cox proportional hazards regression models were used to estimate the associations between antenatal corticosteroid treatment and physician-diagnosed specific developmental disorders of speech and language, scholastic skills, and motor function; pervasive developmental disorder; other or unspecified psychological developmental disorder; disorders of vison and hearing; epilepsy; and cerebral palsy.

**Results:**

The study population comprised 670 097 singleton children (342 562 boys [51.1%]) followed up for a median of 5.8 years (IQR, 3.1-8.7 years). Of the 14 868 treatment-exposed children (2.2%; 53.9% boys), 6730 (45.3%) were born term, and 8138 (54.7%) were born preterm, and of the 655 229 nonexposed children (97.8%; 51.1% boys), 634 757 (96.9%) were born term, and 20 472 (3.1%) were born preterm. Of the 241 621 eligible maternal sibling pairs born term, 4128 (1.7%) were discordant for treatment exposure. Compared with nonexposure in the entire population, treatment exposure was significantly associated with higher adjusted hazard ratios (aHRs) for specific developmental disorders of speech and language (aHR, 1.38 [95% CI, 1.27-1.50]; *P* < .001), specific developmental disorders of scholastic skills (aHR, 1.32 [95% CI, 1.13-1.54]; *P* = .004), specific developmental disorder of motor function (aHR, 1.32 [95% CI, 1.18-1.49]; *P* < .001), pervasive developmental disorder (aHR, 1.35 [95% CI, 1.17-1.56]; *P* < .001), other or unspecified disorder of psychological development (aHR, 1.88 [95% CI, 1.58-2.25]; *P* < .001), and vision or hearing loss (aHR, 1.22 [95% CI, 1.04-1.43]; *P* = .02). Compared with nonexposure in the term-born group, treatment exposure was significantly associated with higher aHRs for specific developmental disorders of speech and language (aHR, 1.47 [95% CI, 1.31-1.66]; *P* < .001), specific developmental disorders of scholastic skills (aHR, 1.28 [95% CI, 1.01-1.63]; *P* = .04), specific developmental disorder of motor function (aHR, 1.38 [95% CI, 1.12-1.70]; *P* < .001), pervasive developmental disorder (aHR, 1.42 [95% CI, 1.16-1.75]; *P* < .001), other or unspecified disorder of psychological development (aHR, 1.92 [95% CI, 1.51-2.43]; *P* < .001), epilepsy (aHR, 1.57 [95% CI, 1.22-2.01]; *P* < .001), and cerebral palsy (aHR, 2.18 [95% CI, 1.47-3.23]; *P* < .001). The hazard for any psychological developmental and neurosensory disorder was significantly higher for the treatment-exposed sibling compared with the nonexposed cosibling (absolute difference, 1.2% [95% CI, 0.03%-2.4%]; *P* < .001; aHR, 1.22 [95% CI, 1.04-1.42]; *P* = .01). Antenatal corticosteroids were not associated with either significant benefit or risk in the preterm group.

**Conclusions and Relevance:**

This study suggests that the possible long-term psychological developmental and neurosensory harms warrant careful consideration of risks and benefits when deciding on maternal antenatal corticosteroid treatment.

## Key Points

**Question:**

Is maternal antenatal corticosteroid treatment associated with psychological developmental and neurosensory disorders in children?

**Findings:**

In this population-based cohort study comprising 670 097 children, exposure to maternal antenatal corticosteroid treatment, compared with nonexposure, was associated with significantly higher rates of psychological developmental and neurosensory disorders in children.

**Meaning:**

The findings suggest that the risks and benefits to the offspring of maternal antenatal corticosteroids should be carefully considered when deciding on treatment.

## Teaser

This population-based cohort study examines whether antenatal maternal corticosteroid treatment is associated with psychological developmental and neurosensory disorders among children born term and preterm and whether the associations persist in a sibling-comparison design.

## Introduction

Corticosteroids administered to women at risk of imminent preterm birth is one of the most effective ways to improve neonatal prognosis of infants born preterm. According to a recent Cochrane meta-analysis of randomized clinical trials (RCTs), robust evidence shows that maternal antenatal corticosteroid treatment (ACT) reduces the child’s risk of perinatal and neonatal mortality and respiratory distress syndrome, and probably also the risk of intraventricular hemorrhage.^1^ Although existing guidelines are consistent in recommending ACT until 34 gestational weeks,^2,3,4^ guidelines differ on whether treatment should be given when late preterm birth is imminent, between 34 weeks and 0 days and 36 weeks and 6 days, or whether treatment should be given at term (≥37 weeks and 0 days) before elective cesarean delivery. In the former situation, ACT prevents respiratory morbidity,^5,6^ although this evidence has been recently challenged.^7^ In the latter situation, ACT reduces the risk of admission to the neonatal intensive care unit (NICU) for respiratory complications.^8^ Antenatal corticosteroid treatment is not associated with mortality in either situation.^5,6,7,8^ The US guidelines recommend consideration of ACT in these situations,^2^ while a similar recommendation was removed from the 2019 update of the European guideline.^3^ The 2021 guideline by the International Federation of Gynaecology and Obstetrics specifically advises against routine use of ACT after 34 weeks and 0 days of gestation.^4^ These extensions would increase the number of children exposed to ACT several fold, as late preterm births alone constitute more than 70% of all preterm births.^9^

Because corticosteroids pass through the placenta and the blood-brain barrier, concerns have been raised that ACT may harm fetal brain development and carry long-term consequences for brain developmental outcomes.^10,11,12,13^ However, the recent Cochrane meta-analysis of RCTs concluded that, among children who were born preterm, ACT appears to carry no such harms and probably leads to a reduced risk of neurodevelopmental delay^1^; moreover, neurosensory function appears unaffected. Another recent meta-analysis of observational studies suggested that, among preterm-born children, a single course of ACT may confer benefits on neurodevelopment and neurosensory function compared with no treatment.^14^ One large population-based study showed that ACT was associated with a higher risk of psychological developmental disorders among exposed children^15^; the risks were mostly due to children who were born at term after the ACT exposure. The mixed pattern of findings may suggest that these factors associated with ACT are not found across all domains of neurodevelopmental and neurosensory function. Moreover, it may also suggest that, while ACT is associated with benefits for children born preterm, who per se are at a higher risk of neurodevelopmental and neurosensory disorders,^16,17^ the risks of ACT exposure are manifest only among the exposed children born at term.^14,15^

Here we extend previous work in 2 ways. First, because psychological developmental disorders were previously studied as a single entity,^15^ here we study whether ACT is associated with specific psychological developmental disorders as outcomes, namely, disorders of speech and language, disorders of scholastic skills, disorders of motor function, pervasive developmental disorder, and other or unspecified psychological developmental disorders in children followed up from birth until 1 to 12 years of age. Second, because the previous study ignored neurosensory disorders, here we also study disorders of vision and hearing, epilepsy, and cerebral palsy (CP) as outcomes. We also examined whether the associations varied by preterm and term birth, and in a sibling-comparison design, we examined whether unmeasured familial confounding explained the associations.

## Methods

### Study Population

With permission, we merged information from different registers kept at the Finnish Institute for Health and Welfare by using unique personal identification numbers assigned to all Finnish citizens and permanent residents. Because the registered individuals were not contacted, Finnish legislation does not require institutional review board evaluation or informed consent. This cohort study followed the Strengthening the Reporting of Observational Studies in Epidemiology (STROBE) reporting guideline.

From the Medical Birth Register, we included all singleton pregnancies with live births in Finland between January 1, 2006, and December 31, 2017.^18^ Since 1987, all live births and stillbirths in Finland with a gestational age of 22 weeks or more or a birth weight of 500 g or more have been included in this register. Infants eligible for data analyses survived until 364 days, had data on gestational age, and had valid maternal and child personal identification codes for register data linkage. From this population, we also identified all consecutive maternal sibling pairs born at term, including sibling pairs in which one was exposed to ACT and the other was not and sibling pairs in which neither sibling was exposed to ACT. The children were followed up from birth until December 31, 2018.

### Maternal ACT

The Medical Birth Register^18^ provides data on maternal ACT (yes or no). Data were not available on the number of treatments or their timing. The Finnish national guidelines^19^ recommended betamethasone, 12 mg, administered twice, 24 hours apart throughout the study period. Until 2009, treatment was recommended until 34 weeks and 0 days of gestation (32 weeks and 0 days in case of premature rupture of membranes); after 2009, treatment was recommended until 34 weeks and 6 days of gestation and, in select cases, later (eg, fetal hydrops or maternal disorder warranting cesarean delivery).^19^ Repeated treatments were not recommended before 2009; after 2009, 1 repeated course could be considered when the risk of respiratory distress was high. As has been previously shown, the treatment recorded in the Medical Birth Register showed high agreement (>97%) with the treatment recorded in patient case reports in 2 clinical cohorts nested within our study population.^15^

### Childhood Psychological Developmental and Neurosensory Disorders

Primary or secondary disorder diagnoses came from the Care Register for Health Care.^20^ Diagnoses were coded using the *International Statistical Classification of Diseases and Related Health Problems, Tenth Revision* (*ICD-10*), comprising all hospital inpatient treatments since 1969 and outpatient treatments since 1998 by physicians in specialized medical care. This register has high validity for psychiatric diagnoses.^20,21^ The primary outcomes were specific developmental disorders of speech and language (*ICD-10* code F80), scholastic skills (*ICD-10* code F81), and motor function (*ICD-10* code F83); pervasive developmental disorder (*ICD-10* code F84); other or unspecified psychological development disorders (*ICD-10* codes F88 and F89); disorders of vision and hearing (*ICD-10* codes H54, H90, and H91); epilepsy (*ICD-10* codes G40 and G41); and CP (*ICD-10* code G80).

### Covariates

We identified covariates that have previously been shown to be associated with ACT, preterm birth, and/or psychological developmental or neurosensory disorders.^2,3,4,16,17,22^ We identified these covariates from the Medical Birth Register,^18^ including the child’s birth year, sex, 1-minute and 5-minute Apgar score, admission to the NICU, weight and gestational age at birth, and maternal age at delivery, parity, mode of delivery, smoking during pregnancy, prepregnancy body mass index (calculated as weight in kilograms divided by height in meters squared from weight and height verified by measurement in the first antenatal clinic visit between 7 and 10 gestational weeks), premature rupture of membranes (*ICD-10* code O42), gestational diabetes (*ICD-10* code O24), and hypertension in pregnancy (*ICD-10* codes O10 and O13-O15). The Finnish Care Register for Health Care^20^ provided data on maternal diagnoses of mental and behavioral disorders (*ICD-10* codes F00-F99); eye, adnexa, ear, and mastoid disorders (*ICD-10* codes H00-H95); and nervous system disorders (*ICD-10* codes G00-G99) between 1996 and December 31, 2018. In sensitivity analyses, we excluded children with major congenital anomalies with diagnoses coming from the Register of Congenital Anomalies according to EUROCAT inclusion criteria.^23^ Race and ethnicity are not recorded in Finnish registers owing to European Union legislation.

### Statistical Analysis

Statistical analysis was performed from March 21, 2021, to July 7, 2022. We used Cox proportional hazards regression models to estimate the associations between ACT exposure and psychological developmental and neurosensory disorders in children. We conducted the analyses in the entire cohort and among term-born and preterm-born children. We used Kaplan-Meier curves to estimate the time from birth to the first diagnosis of the outcomes and the cumulative probability to remain diagnosis free at the end of the follow-up.

To compare term-born siblings discordant for ACT exposure (treatment exposed − nonexposed and nonexposed − treatment exposed), we used stratified Cox proportional hazards regression models, with each set of siblings representing separate strata. For these analyses we used any psychological developmental and neurosensory disorder as the primary outcome because of the lack of sufficient statistical power to reliably estimate the hazard of each of the specific disorders. Because of secular trends in seeking health care,^24^ we also compared sibling pairs in which the younger child was exposed to ACT and the older child was not exposed with a sibling pair in which both siblings were not exposed. To account for the dependence of sibling observations in our analyses, we compared the first set of siblings for each mother.

We present the associations as unadjusted and adjusted for all covariates. In sensitivity analyses, we excluded children with major congenital anomalies. The sibling comparisons were adjusted for maternal age at delivery, smoking during pregnancy, parity, and child’s sex and birth year, and in the sibling comparisons addressing secular trends, we made further adjustments for any psychological developmental or neurosensory disorder of the older sibling who was not exposed to ACT.

As effect sizes, we reported absolute differences in cumulative incidence rates and hazard ratios (HRs) with 95% CIs. We regard 2-sided *P* < .05 as statistically significant. Proportional hazards assumptions, verified as acceptable, were assessed on the plots of log (time) vs log (−log [survival]) and using Schoenfeld tests. Plots of deviance and Martingale residuals detected no outliers or nonlinearity. We conducted complete-case analyses because missing data in our study population were minimal (Table; eTable 1 in the Supplement), except for smoking (<4.1%), for which missing values were treated as a separate category. We performed all statistical analyses using SAS, version 9.4 (SAS Institute Inc).

**Table.** Characteristics of the Entire Cohort of Children and Children Born at Term (≥37 Gestational Weeks) and Preterm (<37 Gestational Weeks) According to Maternal Antenatal Corticosteroid Treatment Exposure

Characteristic	Children, No. (%)					
	Entire cohort (N = 670 097)		Term (n = 641 487)		Preterm (n = 28 610)	
	Treatment-exposed	Nonexposed	Treatment-exposed	Nonexposed	Treatment-exposed	Nonexposed
Total No.^a^	14 868	655 229	6730	634 757	8138	20 472
**Children**						
Sex						
Boy	8010 (53.9)	334 552 (51.1)	3513 (52.2)	323 014 (50.9)	4497 (55.3)	11 538 (56.4)
Girl	6858 (46.1)	320 677 (48.9)	3217 (47.8)	311 743 (49.1)	3641 (44.7)	8934 (43.6)
Gestational age at birth, mean (SD), wk	35.8 (4.0)	39.9 (1.3)	39.3 (1.3)	40.1 (1.2)	32.8 (3.0)	35.5 (1.7)
Birth weight, mean (SD), g	2659 (950)	3548 (496)	3409 (510)	3577 (465)	2038 (764)	2659 (603)
Apgar score (maximum of 1 and 5 min)^b^						
0-3	192 (1.3)	1172 (0.2)	12 (0.2)	985 (0.2)	180 (2.2)	187 (0.9)
4-6	1056 (7.1)	8495 (1.3)	104 (1.6)	7514 (1.2)	952 (11.7)	981 (4.8)
7-10	13 504 (90.8)	644 722 (98.4)	6598 (98.0)	625 512 (98.5)	6906 (84.9)	19 210 (93.8)
Unknown	116 (0.8)	840 (0.1)	16 (0.2)	746 (0.1)	100 (1.2)	94 (0.5)
Admission to NICU						
No	7360 (49.5)	594 363 (90.7)	5884 (87.4)	583 969 (92.0)	1476 (18.1)	10 394 (50.8)
Yes	7508 (50.5)	60 866 (9.3)	846 (12.6)	50 788 (8.0)	6662 (81.9)	10 078 (49.2)
Major congenital anomaly						
No	13 280 (89.3)	625 583 (95.5)	6279 (93.3)	606 919 (95.6)	7001 (86.0)	18 664 (91.2)
Yes	1588 (10.7)	29 646 (4.5)	451 (6.7)	27 838 (4.4)	1137 (14.0)	1808 (8.8)
**Mothers**						
Age at delivery, mean (SD), y	30.6 (5.8)	30.3 (5.3)	30.1 (5.8)	30.3 (5.4)	31.0 (5.7)	30.4 (5.7)
Parity						
0	6750 (45.4)	271 889 (41.5)	2758 (41.0)	261 720 (41.2)	3992 (49.1)	10 169 (49.7)
1	4458 (30.0)	222 606 (34.0)	2287 (34.0)	216 966 (34.2)	2171 (26.7)	5640 (27.5)
2	2081 (14.0)	95 989 (14.6)	1004 (14.9)	93 469 (14.7)	1077 (13.2)	2520 (12.3)
3	796 (5.4)	33 078 (5.0)	369 (5.5)	31 991 (5.0)	427 (5.2)	1087 (5.3)
≥4	783 (5.3)	31 561 (4.8)	312 (4.6)	30 508 (4.8)	471 (5.8)	1053 (5.1)
Unknown	0	106 (0.02)	0	103 (0.0)	0	3 (0.0)
Delivery mode						
Vaginal	9324 (62.7)	554 481 (84.6)	5471 (81.3)	540 301 (85.1)	3853 (47.3)	14 180 (69.3)
Cesarean	5544 (37.3)	100 748 (15.4)	1259 (18.7)	94 456 (14.9)	4285 (52.7)	6292 (30.7)
Prepregnancy BMI, mean (SD)	24.5 (5.4)	24.4 (4.9)	24.1 (5.3)	24.4 (4.9)	24.9 (5.5)	24.6 (5.2)
Unknown	161 (1.1)	13 076 (2.0)	52 (0.8)	12 428 (2.0)	109 (1.3)	648 (3.2)
Premature rupture of membranes^c^						
No	12 689 (83.3)	640 200 (97.3)	6502 (96.6)	620 821 (97.8)	5917 (72.7)	17 046 (83.3)
Yes	2538 (16.7)	17 451 (2.7)	228 (3.4)	13 936 (2.2)	2221 (27.3)	3426 (16.7)
Gestational diabetes^c^						
No	1234 (83.2)	581 503 (88.7)	5666 (84.2)	564 572 (89.9)	6708 (82.7)	16 931 (82.4)
Yes	2494 (16.8)	73 726 (11.3)	1064 (15.8)	70 185 (11.1)	1430 (17.3)	3541 (17.6)
Hypertension^c^						
No	13 335 (89.7)	629 030 (96.0)	6340 (94.2)	610 711 (96.2)	6995 (86.0)	18 319 (89.5)
Yes	1533 (10.3)	26 199 (4.0)	390 (5.8)	24 046 (3.8)	1143 (14.0)	2153 (10.5)
Any mental or behavioral disorder^c^						
No	10 895 (73.3)	534 540 (81.6)	4854 (72.1)	518 694 (81.7)	6041 (74.2)	15 846 (77.4)
Yes	3973 (26.7)	120 689 (18.4)	1876 (27.9)	116 063 (18.3)	2097 (25.8)	4626 (22.6)
Any eye, adnexa, ear, or mastoid disorder^c^						
No	14 868 (89.0)	611 301 (93.3)	6730 (89.9)	634 757 (93.3)	8138 (88.3)	20 472 (91.7)
Yes	1633 (11.0)	44 198 (6.7)	682 (10.1)	42 497 (6.7)	951 (11.7)	1701 (8.3)
Any nervous system disorder^c^						
No	13 590 (91.4)	620 564 (94.7)	6184 (91.9)	601 472 (94.8)	7406 (91.0)	19 092 (93.3)
Yes	1278 (8.6)	34 665 (5.3)	546 (8.1)	33 285 (5.2)	732 (9.0)	1380 (6.7)
Smoking during pregnancy						
No	11 717 (78.8)	543 002 (82.8)	5316 (79.0)	526 906 (83.0)	6401 (78.7)	16 096 (78.6)
Yes	2767 (18.6)	95 883 (14.6)	1301 (19.3)	92 337 (14.5)	1466 (18.0)	3546 (17.3)
Unknown	384 (2.6)	16 344 (2.5)	113 (1.7)	15 514 (2.4)	271 (3.3)	830 (4.1)

Abbreviations: BMI, body mass index (calculated as weight in kilograms divided by height in meters squared); NICU, neonatal intensive care unit.

^a^ Percentages may not total 100% owing to rounding.

^b^ The Apgar score is calculated at 1 and 5 minutes after birth and uses skin color, heart rate, reflexes, muscle tone, and respiratory effort to determine medical attention: scores 0 to 3 suggest a need for resuscitation, while scores of 7 or more are considered normal.

^c^ *International Statistical Classification of Diseases and Related Health Problems, Tenth Revision* codes: premature rupture of membranes, O42; gestational diabetes, O24; hypertension, O10, O13-O15; any mental or behavioral disorder, F00-F99; any eye, adnexa, ear, or mastoid disorder, H00-H95; and any nervous system disorder, G00-G99.

## Results

The Table shows child and maternal characteristics of the entire eligible treatment-exposed and nonexposed study population (N = 670 097; 342 562 boys [51.1%]). Of the 14 868 treatment-exposed children (2.2%; 53.9% boys), 6730 (45.3%) were born at term, and 8138 (54.7%) were born preterm. Of the 655 229 nonexposed children (97.8%; 51.1% boys), 634 757 (96.9%) were born at term, and 20 472 (3.1%) were born preterm. Compared with the nonexposed children, treatment-exposed children in the entire study population were born earlier in gestation (mean [SD], 35.8 [4.0] vs 39.9 [1.3] weeks), had lower birth weights (mean [SD], 2659 [950] vs 3548 [496] g), were more often admitted to the NICU (50.5% vs 9.3%), more often received a diagnosis of major congenital anomaly (10.7% vs 4.5%), and were more often delivered via cesarean birth (37.3% vs 15.4%). Mothers of treatment-exposed children were more often primiparous (45.4% vs 41.5%); had premature rupture of membranes (16.7% vs 2.7%); had gestational diabetes (16.8% vs 11.3%); had hypertension in pregnancy (10.3% vs 4.0%); had any mental or behavioral disorder (26.7% vs 18.4%); had any eye, adnexa, ear, or mastoid disorder (11.0% vs 6.7%); had any nervous system disorder (8.6% vs 5.3%); and were more likely to have smoked during pregnancy (18.6% vs 14.6%). Distributions of children’s sex (53.9% vs 51.1% boys), maternal age at delivery (mean [SD], 30.6 [5.8] vs 30.3 [5.8] years), and maternal prepregnancy body mass index (mean [SD], 24.5 [5.4] vs 24.4 [4.9]) were similar between the treatment-exposed and nonexposed groups. The children were followed up a median of 5.8 years (IQR, 3.1-8.7 years).

### Psychological Developmental and Neurosensory Disorders in Treatment-Exposed and Nonexposed Children

The Figure shows the unadjusted cumulative incidence rates, and eTable 2 in the Supplement shows the median age and IQR at the first diagnosis of psychological developmental and neurosensory disorders for the treatment-exposed and nonexposed children in the entire cohort and in the groups born at term and preterm. Adjusted HRs from multivariable models showed that, in the entire cohort, the treatment-exposed children had significantly higher adjusted hazard ratios (aHRs) than the nonexposed children for specific developmental disorders of speech and language (absolute difference, 2.6% [95% CI, 2.2%-2.9%]; *P* < .001; aHR, 1.38 [95% CI, 1.27-1.50]; *P* < .001), specific developmental disorders of scholastic skills (absolute difference, 0.6% [95% CI, 0.4%-0.8%]; *P* < .001; aHR, 1.32 [95% CI, 1.13-1.54]; *P* = .004), specific developmental disorder of motor function (absolute difference, 2.7% [95% CI, 2.4%-3.0%]; *P* < .001; aHR, 1.32 [95% CI, 1.18-1.49]; *P* < .001), pervasive developmental disorder (absolute difference, 0.6% [95% CI, 0.4%-0.8%]; *P* < .001; aHR, 1.35 [95% CI, 1.17-1.56]; *P* < .001), other or unspecified disorder of psychological development (absolute difference, 0.5% [95% CI, 0.3%-0.7%]; *P* < .001; aHR, 1.88 [95% CI, 1.58-2.25]; *P* < .001), and vision or hearing loss (absolute difference, 0.9% [95% CI, 0.7%-1.1%]; *P* < .001; aHR, 1.22 [95% CI, 1.04-1.43]; *P* = .02) (Figure). In the term-born group, compared with nonexposure, treatment exposure was significantly associated with higher aHRs for specific developmental disorders of speech and language (absolute difference, 1.3% [95% CI, 0.8%-1.8%]; *P* < .001; aHR, 1.47 [95% CI, 1.31-1.66]; *P* < .001), specific developmental disorders of scholastic skills (absolute difference, 0.1% [95% CI, –0.1% to 0.7%]; *P* = .31; aHR, 1.28 [95% CI, 1.01-1.63]; *P* = .04), specific developmental disorder of motor function (absolute difference, 0.5% [95% CI, 0.2%-0.7%]; *P* < .001; aHR, 1.38 [95% CI, 1.12-1.70]; *P* < .001), pervasive developmental disorder (absolute difference, 0.4% [95% CI, 0.1%-0.6%]; *P* = .004; aHR, 1.42 [95% CI, 1.16-1.75]; *P* < .001), other or unspecified disorder of psychological development (absolute difference, 0.5% [95% CI, 0.2%-0.7%]; *P* < .001; aHR, 1.92 [95% CI, 1.51-2.43]; *P* < .001), epilepsy (absolute difference, 0.4% [95% CI, 0.1%-0.6%]; *P* < .001; aHR, 1.57 [95% CI, 1.22-2.01]; *P* < .001), and cerebral palsy (absolute difference, 0.2% [95% CI, 0.0%-0.4%]; *P* < .001; aHR, 2.18 [95% CI, 1.47-3.23]; *P* < .001). In the preterm group, treatment-exposed and nonexposed children did not differ significantly in the hazards of any of the disorders in the multivariable models adjusted for the covariates.

**Figure.**
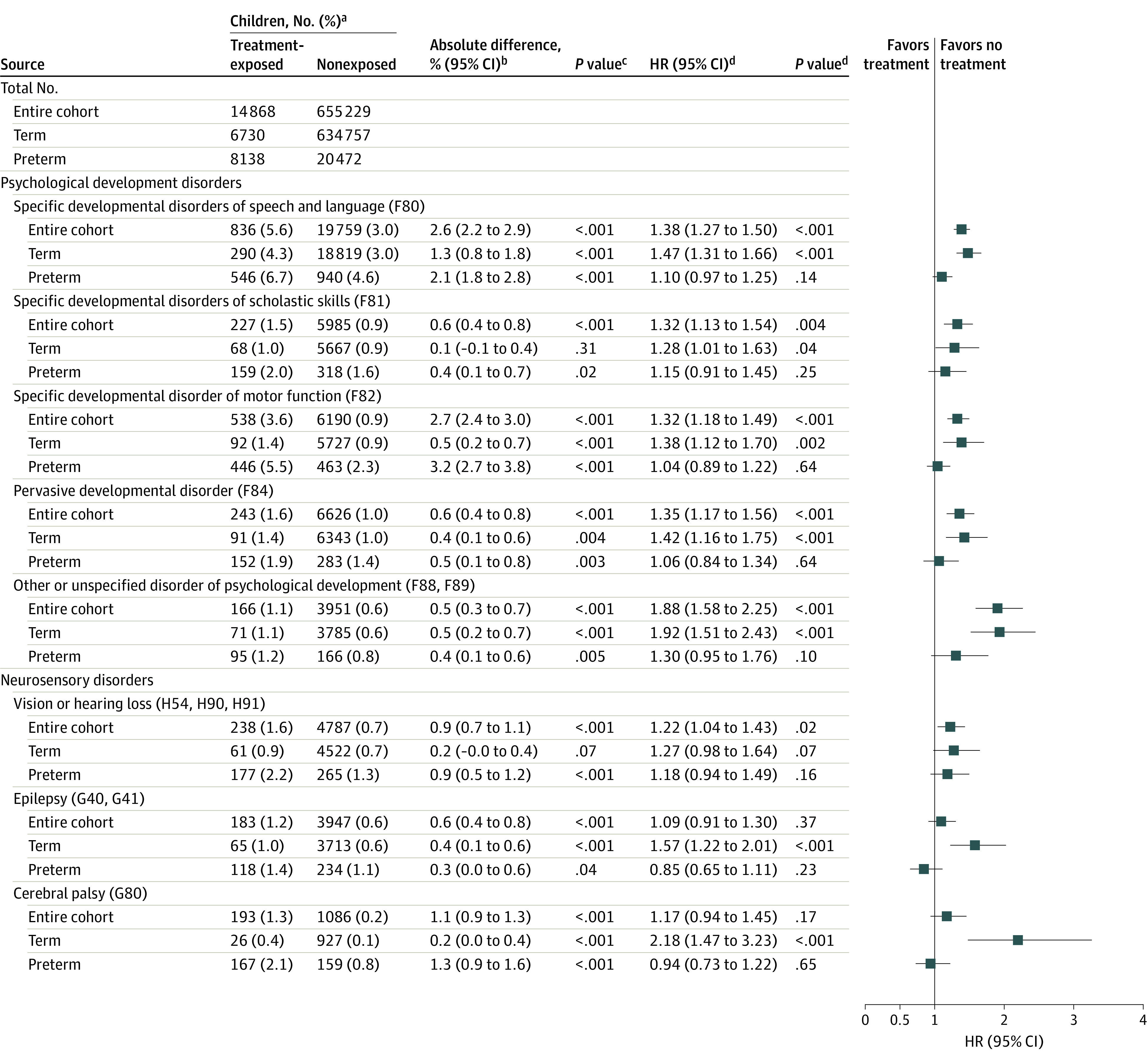
Unadjusted Cumulative Incidence Rates and Adjusted Hazard Ratios (HRs) of Psychological Developmental and Neurosensory Disorders in Children According to Maternal Antenatal Corticosteroid Treatment Exposure ^a^Number of children with diagnosis of psychological developmental and neurosensory disorders and cumulative incidences during follow-up of the entire cohort of children (unadjusted analyses, 670 097; adjusted analyses, 642 158), term-born children (unadjusted analyses, 641 487; adjusted analyses, 615 305), and preterm-born children (unadjusted analyses, 28 610; adjusted analyses, 26 853) eligible for data analyses. ^b^Absolute differences may differ from the arithmetic difference of group totals because of rounding. ^c^From χ^2^ statistics. ^d^Hazard ratios, 95% CIs, and *P* values are from multivariable Cox proportional hazards regression models adjusted for maternal age at delivery, parity, mode of delivery, maternal smoking during pregnancy, prepregnancy body mass index, premature rupture of membranes (*International Statistical Classification of Diseases and Related Health Problems, Tenth Revision* [*ICD-10*] code O42), gestational diabetes (*ICD-10* code O24), hypertension in pregnancy (*ICD-10* codes O10 and O13-O15), and child sex, Apgar score (maximum of 1 and 5 min), admission to neonatal intensive care unit, weight and gestational age at birth. For child psychological developmental disorders, models were adjusted additionally for maternal mental and behavioral disorder diagnoses (*ICD-10* codes F00-F99); for child vision and hearing disorders, models were adjusted additionally for maternal eye, adnexa, ear, and mastoid disorder diagnoses (*ICD-10* codes H00-H95); and for child epilepsy and cerebral palsy, models were adjusted additionally for maternal nervous system disorder diagnoses (*ICD-10* codes G00-G99).

eTable 3 in the Supplement shows that when we excluded children with major congenital anomalies (31 234 [4.7%] of the entire cohort; 28 289 [4.4%] in the term group and 2945 [10.3%] in the preterm group) from the analyses, the aHRs changed only in 2 cases: in the entire cohort, the higher aHR for CP among the treatment-exposed compared with nonexposed children became significant; for the term-born children, the higher aHR for specific disorders of scholastic skills for the treatment-exposed compared with the nonexposed children was rendered nonsignificant.

### Term-Born Sibling Comparisons

Our eligible sibling sample comprised 241 621 consecutive maternal term-born sibling pairs, of whom 4128 were discordant for treatment exposure. The aHR for any psychological developmental and neurosensory disorder was significantly higher for the treatment-exposed compared with the nonexposed sibling (8.4% vs 7.2%; absolute difference, 1.2% [95% CI, 0.03%-2.4%]; *P* < .001; aHR, 1.22 [95% CI, 1.04-1.42]; *P* = .01). The aHR was also significantly higher for the younger treatment-exposed sibling of the treatment-exposure discordant sibling pair (n = 2141) compared with the younger nonexposed sibling of the nonexposure concordant sibling pair (n = 237 319) (6.9% vs 4.7%; absolute difference, 2.2% [95% CI, 1.1%-3.3%]; *P* < .001; aHR, 1.33 [95% CI, 1.17-1.51]; *P* < .001).

### Exploratory Post Hoc Analyses

Because the Finnish national treatment regimen of maternal ACT changed in 2009,^19^ we conducted analyses in 2 strata: the entire cohort of children born during the period from 2006 to 2008 and those born during the period from 2009 to 2017. eTable 4 in the Supplement shows that the aHRs were similar in the 2 strata, except for specific disorders of scholastic skills and vision or hearing loss, for which the associations were not statistically significant among the younger children.

## Discussion

This large population-based cohort study showed that ACT was associated with long-term neurodevelopmental and neurosensory harms among the treatment-exposed children compared with the nonexposed children followed up from birth until age 1 to 12 years. Excess risk of these harms appeared to be nonspecific to the domain of neurodevelopment and neurosensory function and manifested in the children who, after treatment exposure, were born at term. In the entire cohort and among the term-born treatment-exposed children, who comprised nearly half the treatment-exposed children, the HRs were significantly higher for specific disorders of speech and language, scholastic skills, and motor function, as well as for pervasive and other unspecified disorders of psychological development. Moreover, in the entire cohort, the HR was also significantly higher for vision and hearing disorders, and among the term-born children, the HR was significantly higher for epilepsy and CP. These higher HRs were not explained by important mother- and child-related covariates and changed only a little when we excluded children with major congenital anomalies from the analyses.

Although the preterm treatment-exposed children had significantly higher cumulative incidence rates of all psychological developmental and neurosensory disorders than the nonexposed preterm children, in multivariable models, none of the aHRs were statistically significant after adjusting for mother- and child-related covariates. This finding suggests that the risks associated with preterm birth seemed to outweigh any additional risks associated with ACT in this group. This result may, however, also suggest that ACT does not appear to be associated with long-term neurodevelopmental or neurosensory benefits for the preterm children; regardless of treatment exposure, they had higher cumulative incidence rates for all psychological developmental and neurosensory disorders, even when compared with the children born at term after treatment exposure.

The term-born sibling comparisons showed that familial factors shared by siblings did not explain the associations because the treatment-exposed term-born sibling had a significantly higher HR for any psychological developmental and neurosensory disorder than the nonexposed term-born cosibling. The term-born sibling comparisons also showed that the associations were not explained by secular trends (ie, higher likelihood for parents to seek care for their younger children for these disorders)^24^ because the younger treatment-exposed sibling in the treatment exposure–discordant sibling pair had a significantly higher HR for any psychological developmental and neurosensory disorder than the younger nonexposed sibling in the nonexposure-concordant sibling pair.

These findings lend credence to the guidelines, which limit administration of ACT until 34 gestational weeks^3,4^; the 2.3% to 4.3% lower rates of perinatal and neonatal mortality and respiratory distress syndrome among the treatment-exposed children^1^ outweigh the 0.5% to 2.6% higher rates of long-term psychological developmental and neurosensory disorders reported here. However, these long-term harms may call into question the benefits associated with ACT administered in the late preterm window and at term before an elective cesarean delivery. Even the benefits associated with ACT administered in the late preterm window are controversial; a meta-analysis of 7 RCTs, with evidence judged as low to high in certainty, has shown that ACT administered from 34 weeks and 0 days to 36 weeks and 6 days of gestation decreased the risk of respiratory morbidity but, at the same time, increased the risk for neonatal hypoglycemia.^6^ Another RCT that also administered ACT from 34 weeks and 0 days to 36 weeks and 6 days of gestation showed no benefits of ACT for perinatal or neonatal morbidity or mortality and was stopped because of a lower-than-expected prevalence of primary outcomes and slow recruitment.^7^ Moreover, the benefits of ACT administered at term before an elective cesarean delivery are questionable and based on evidence judged as low or very low in certainty, with only 1 RCT suggesting, with a moderate degree of certainty, that ACT probably decreases the risk of admission to the NICU for respiratory complications.^8^

Our findings challenge the Cochrane meta-analysis,^1^ including 3 RCTs with neurodevelopmental delay as an outcome. These RCTs included a total of 600 children aged 2 to 12 years, born preterm in the 1980s or 1990s, and suggested with a moderate degree of certainty that ACT was associated with a reduced risk of neurodevelopmental delay. Our findings are also in disagreement with a meta-analysis of 2 observational studies of more than 5000 children aged 18 to 22 months, born preterm during the period from 2000 to 2011, that suggested with a low degree of certainty that ACT was associated with a reduced risk of a composite outcome comprising delays in cognitive, language, or motor development; CP; or vision or hearing loss.^14^ However, none of the studies included in these meta-analyses included children who, after treatment exposure, were born at term; hence, those studies were not able to compare outcomes between treatment-exposed and nonexposed children born at term and preterm. Two previous studies with a focus on neurodevelopment have done so, but one study compared term-born children who were exposed to multiple courses of ACT with term-born peers exposed to a single course of ACT,^25^ and the other study included suspected neurodevelopmental and neurosensory problems in the outcome, increasing the number of children with problems multifold above the true incidence rates.^26^ However, the suspected neurodevelopmental and neurosensory problems in the latter study^26^ likely included children whose problems were milder and not captured by diagnosed disorders, suggesting that ACT for term-born children may be associated with harms across a wide spectrum of symptomatic severity.

### Limitations

Our study has some limitations. We cannot draw causal inferences or rule out residual confounding. Even though our eligible study population comprised all births in Finland surviving to age 1 year, individual-level data on deaths were not available. However, the effect of this bias is minimal because national vital statistics show that 0.2 per 1000 children were expected to have died after infancy during the follow-up.^27^ Furthermore, even though our sample was large (ie, comprising the entire population), we still had limited statistical power in comparisons of treatment-exposed and nonexposed children and could not compare preterm-born sibling pairs. Because we studied only births in Finland, generalizations to other populations are limited. Because the timing of ACT and the number and types of treatments given are not recorded in the Medical Birth Register, we were not able to study whether the associations varied accordingly. We focused on physician diagnoses of psychological developmental and neurosensory disorders made in primary care hospitals and outpatient clinics in specialized medical care. Hence, our study is likely to capture more severe disorders, and our sample may comprise children with disorders diagnosed in other settings. However, this focus may have attenuated rather than accelerated our ability to detect significant associations. During the study period, minor changes were made to the treatment regimen for maternal ACT in Finland.^19^ The associations changed only a little when we conducted the analyses among the children born before and after this change. Rather than reflecting changes in the treatment regimen, any differences may reflect differences between the children at the median age at the first-disorder diagnosis and during follow-up because children born before this change were followed up from birth until 10 to 12 years of age, while those born after the change were followed up from birth until age 1 to 9 years. Finally, we were unable to assess the potential mechanisms mediating the association between ACT and psychological developmental and neurosensory disorders. Fetal hypoglycemia, which is associated with ACT^5,6^ and neurodevelopmental delays in children,^28^ could be a contributing factor because it may lie on the same pathway.

## Conclusions

In this population-based cohort study, exposure to maternal ACT was significantly associated with higher HRs for psychological developmental and neurosensory disorders in children. These findings warrant careful consideration of risks and benefits when deciding on maternal ACT.

## Notes

Supplement. **eTable 1.** Comparison of Children and Mothers With Complete and Missing Data **eTable 2.** Median Age and Interquartile Range (IQR) at First Diagnosis of Psychological Developmental and Neurosensory Disorders in Children According to Maternal Antenatal Corticosteroid Treatment Exposure **eTable 3.** Adjusted Hazard Ratios (HR) of Psychological Developmental and Neurosensory Disorders in Children Without Major Congenital Anomalies According to Maternal Antenatal Corticosteroid Treatment Exposure **eTable 4.** Adjusted Hazard Ratios (HR) of Psychological Developmental and Neurosensory Disorders in Children Born in 2006-2008 and 2009-2017 According to Maternal Antenatal Corticosteroid Treatment Exposure
