# Supplementary material for: Associations Between Maternal Antenatal Corticosteroid Treatment and Psychological Developmental and Neurosensory Disorders in Children
Source: JAMA Netw Open. 2022 Aug 24;5(8):e2228518. doi: 10.1001/jamanetworkopen.2022.28518 (PMC9403777; doi:10.1001/jamanetworkopen.2022.28518)
Supplement: Supplement. — eTable 1. Comparison of Children and Mothers With Complete and Missing Data eTable 2. Median Age and Interquartile Range (IQR) at First Diagnosis of Psychological Developmental and Neurosensory Disorders in Children According to Maternal Antenatal Corticosteroid Treatment Exposure eTable 3. Adjusted Hazard Ratios (HR) of Psychological Developmental and Neurosensory Disorders in Children Without Major Congenital Anomalies According to Maternal Antenatal Corticosteroid Treatment Exposure eTable 4. Adjusted Hazard Ratios (HR) of Psychological Developmental and Neurosensory Disorders in Children Born in 2006-2008 and 2009-2017 According to Maternal Antenatal Corticosteroid Treatment Exposure [file jamanetwopen-e2228518-s001.pdf]

Supplementary Online Content

Räikkönen K, Gissler M, Tapiainen T, Kajantie E. Associations between maternal antenatal corticosteroid treatment and psychological developmental and neurosensory disorders in children. *JAMA Netw Open.* 2022;5(8):e2228518.
doi:10.1001/jamanetworkopen.2022.28518

eTable 1. Comparison of Children and Mothers With Complete and Missing Data
eTable 2. Median Age and Interquartile Range (IQR) at First Diagnosis of Psychological Developmental and Neurosensory Disorders in Children According to Maternal Antenatal Corticosteroid Treatment Exposure
eTable 3. Adjusted Hazard Ratios (HR) of Psychological Developmental and Neurosensory Disorders in Children Without Major Congenital Anomalies According to Maternal Antenatal Corticosteroid Treatment Exposure
eTable 4. Adjusted Hazard Ratios (HR) of Psychological Developmental and Neurosensory Disorders in Children Born in 2006-2008 and 2009-2017 According to Maternal Antenatal Corticosteroid Treatment Exposure

This supplementary material has been provided by the authors to give readers additional information about their work.

© 2022 Räikkönen K et al. *JAMA Network Open.*

**eTable 1.** Comparison of Children and Mothers With Complete and Missing Data			
	**Entire cohort N=670097**		
**Characteristics**	**Complete data N=642158[a]**	**Missing data N=27939[a]**	***P***
**Children:**			
Maternal antenatal corticosteroid treatment, no. %			
Treatment-exposed	14229 (2.2)	639 (2.3)	.43
Nonexposed	627929 (97.8)	27300 (97.7)	
Sex, no. (%)			
Boy	328391 (51.1)	14171 (50.7)	.17
Girl	313767 (48.9)	13768 (49.3)	
Gestational age at birth, mean (SD), weeks	39.8 (1.6)	39.6 (2.0)	<.001
Birth weight, mean (SD), g	3530 (525)	3496 (567)	<.001
Apgar score (maximum of 1 and 5 minutes)[b]			
0-3	9683 (1.5)	477 (1.7)	.004
4-6	27292 (4.3)	1117 (4.0)	
7-10	605183 (94.2)	26345 (94.3)	
Admission to neonatal intensive care unit, no. (%)			
No	576991 (89.9)	24732 (88.5)	<.001
Yes	65167 (10.1)	3207 (11.5)	
Major congenital anomaly, no. (%)			
No	612314 (95.4)	26549 (95.0)	<.001
Yes	29844 (4.6)	1390 (5.0)	
**Mothers:**			
Age at delivery, mean (SD), years	30.3 (5.3)	30.2 (5.6)	.06
Parity, no. (%)			
0	268681 (41.8)	9958 (35.6)	<.001
1	217305 (33.8)	9759 (34.9)	
2	93427 (14.5)	4643 (16.6)	
3	32095 (5.0)	1779 (6.4)	
≥4	30650 (4.8)	1800 (6.4)	
Delivery mode, no. (%)			
Vaginal	540682 (84.2)	23123 (82.8)	<.001
Caesarean	101476 (15.8)	4816 (17.2)	
Pre-pregnancy body mass index, mean (SD), kg/m^2	24.4 (4.9)	24.7 (5.1)	<.001
Premature rupture of membranes, no. (%)[c]			
No	622971 (97.0)	27315 (97.8)	<.001
Yes	19187 (3.0)	624 (2.2)	
Gestational diabetes, no. (%)[c]			

© 2022 Räikkönen K et al. *JAMA Network Open.*

No	568889 (88.6)	24988 (89.4)	<.001
Yes	73269 (11.4)	2951 (10.6)	
Hypertension, no. (%)[c]			
No	615609 (95.9)	26756 (95.8)	.41
Yes	26549 (4.1)	1183 (4.2)	
Any mental or behavioral disorder, no. (%)[c]			
No	523307 (81.5)	22128 (79.2)	<.001
Yes	118851 (18.5)	5811 (20.8)	
Any eye, adnexa, ear or mastoid disorder, no. (%)[c]			
No	598360 (93.2)	25906 (92.7)	.003
Yes	43798 (6.8)	2033 (7.3)	
Any nervous system disorder, no. (%)[c]			
No	607857 (94.7)	26297 (94.1)	<.001
Yes	34301 (5.3)	1642 (5.9)	
Smoking during pregnancy, no. (%)[c]			
No	545151 (84.9)	26296 (94.1)	<.001
Yes	97007 (15.1)	1643 (5.9)	

[a]Percentages may not total up to 100% due to rounding.
[b]The Apgar score is calculated at 1 and 5 minutes after birth, uses skin color, pulse rate, reflexes, muscle tone, and respiratory effort to determine medical attention: Scores 0-3 suggest a need for resuscitation, while scores of 7 or more are considered normal.
[c]International Statistical Classification of Diseases and Related Health problems, tenth revision codes: Premature rupture of membranes, O42; Gestational diabetes, O24; Hypertension, O10, O13-O15; Any mental or behavioral disorder, F00-F99; Any eye, adnexa, ear, or mastoid disorder, H00-H95; Any nervous system disorder, G00-G99.

© 2022 Räikkönen K et al. *JAMA Network Open.*

eTable 2. Median Age and Interquartile Range (IQR) at First Diagnosis of Psychological Developmental and Neurosensory Disorders in Children According to Maternal Antenatal Corticosteroid Treatment Exposure

	**Entire cohort N=670097**		**Term N=641487**		**Preterm N=28610**	
	Treatment-exposed N=14868	Nonexposed N=65522	Treatment-exposed N=6730	Nonexposed N=634757	Treatment-exposed N=8138	Nonexposed N=20472
**Outcome**	Median (IQR)	Median (IQR)	Median (IQR)	Median (IQR)	Median (IQR)	Median (IQR)
**Psychological developmental disorders**						
Specific developmental disorders of speech and language (F80)	4.2 (3.2 to 5.4)	4.6 (3.7 to 5.9)	4.6 (3.5 to 5.8)	4.6 (3.7 to 5.9)	4.0 (3.1 to 5.2)	4.3 (3.2 to 5.6)
Specific developmental disorders of scholastic skills (F81)	8.5 (6.9 to 10.1)	9.1 (7.2 to 10.6)	9.2 (7.8 to 10.8)	9.1 (7.6 to 10.6)	8.4 (6.5 to 9.6)	9.1 (7.0 to 10.4)
Specific developmental disorder of motor function (F82)	3.7 (1.0 to 5.3)	5.1 (3.2 to 6.3)	4.9 (2.3 to 6.3)	5.1 (3.4 to 6.3)	3.3 (0.9 to 5.1)	4.5 (1.6 to 5.9)
Pervasive developmental disorder (F84)	5.7 (3.3 to 8.6)	6.8 (4.0 to 9.5)	6.5 (4.1 to 8.8)	6.9 (4.0 to 9.6)	4.5 (3.0 to 8.1)	6.6 (3.9 to 9.0)
Other or unspecified disorder of psychological development (F88, F89)	6.5 (4.5 to 8.9)	7.3 (5.6 to 9.5)	6.3 (5.0 to 8.2)	7.4 (5.6 to 9.5)	6.5 (4.3 to 9.0)	6.7 (4.8 to 9.0)

© 2022 Räikkönen K et al. *JAMA Network Open.*

**Neurosensory disorders**						
Vision or hearing loss (H54, H90, H91)	3.6 (0.9 to 6.2)	5.2 (2.1 to 6.8)	4.3 (1.1 to 6.4)	5.2 (2.2 to 6.9)	3.6 (0.9 to 6.0)	4.1 (1.2 to 6.3)
Epilepsy (G40, G41)	2.1 (0.7 to 4.3)	3.0 (1.0 to 5.7)	2.2 (0.7 to 4.3)	3.0 (1.0 to 5.8)	2.1 (0.7 to 4.2)	2.3 (0.6 to 4.9)
Cerebral palsy (G80)	2.0 (1.2 to 3.6)	1.9 (1.0 to 3.6)	3.2 (1.6 to 4.6)	1.9 (1.0 to 3.8)	2.0 (1.1 to 3.5)	1.6 (0.9 to 2.6)
F-, G-, and H-codes in parenthesis refer to International Statistical Classification of Diseases and Related Health Problems, tenth revision diagnostic codes.						

© 2022 Räikkönen K et al. *JAMA Network Open.*

eTable 3. Adjusted Hazard Ratios (HR) of Psychological Developmental and Neurosensory Disorders in Children Without Major Congenital Anomalies According to Maternal Antenatal Corticosteroid Treatment Exposure

	Entire cohort N=638863[a]		Term N=613198[a]		Preterm N=25665[a]	
**Outcome**	**Adjusted HR (95% CI)**	***P***	**Adjusted HR (95% CI)**	***P***	**Adjusted HR (95% CI)**	***P***
**Psychological developmental disorders**						
Specific developmental disorders of speech and language (F80)	1.40 (1.28 to 1.53)	<.001	1.48 (1.31 to 1.68)	<.001	1.07 (0.93 to 1.23)	.37
Specific developmental disorders of scholastic skills (F81)	1.29 (1.09 to 1.53)	.003	1.24 (0.96 to 1.61)	.10	1.10 (0.85 to 1.43)	.47
Specific developmental disorder of motor function (F82)	1.35 (1.19 to 1.54)	<.001	1.29 (1.02 to 1.63)	.04	1.03 (0.86 to 1.24)	.74
Pervasive developmental disorder (F84)	1.38 (1.18 to 1.61)	<.001	1.38 (1.11 to 1.73)	.004	1.08 (0.83 to 1.40)	.58
Other or unspecified disorder of psychological development (F88, F89)	1.88 (1.55 to 2.27)	<.001	1.89 (1.47 to 2.43)	<.001	1.37 (0.96 to 1.95)	.08
**Neurosensory disorders**						
Vision or hearing loss (H54, H90, H91)	1.21 (1.00 to 1.48)	.05	1.15 (0.85 to 1.57)	.36	1.07 (0.79 to 1.44)	.69
Epilepsy (G40, G41)	1.14 (0.92 to 1.40)	.23	1.43 (1.07 to 1.92)	.02	0.94 (0.68 to 1.29)	.68
Cerebral palsy (G80)	1.41 (1.09 to 1.82)	.009	2.61 (1.68 to 4.05)	<.001	1.05 (0.78 to 1.44)	.72

[a]In adjusted analyses sample size for the entire cohort of children is 612314, for term-born children 588199, and for preterm-born children 24115.

Adjusted for maternal age at delivery, parity, mode of delivery, maternal smoking during pregnancy, pre-pregnancy body mass index, premature rupture of membranes (O42), gestational diabetes (O24), hypertension in pregnancy (O10, O13-O15), and child sex, Apgar score (maximum of 1 and 5 min), admission to neonatal intensive care unit, weight and gestational age at birth; for child psychological development disorders adjusted additionally for maternal mental and behavioral disorder diagnoses (F00-F99), for child vision and hearing disorders, adjusted additionally for maternal eye, adnexa, ear and mastoid disorder diagnoses (H00-H95), for child epilepsy and cerebral

© 2022 Räikkönen K et al. *JAMA Network Open.*

palsy, adjusted additionally for maternal nervous system disorder diagnoses (G00-G99); F-, G-, H-, and O-codes in parenthesis refer to International Statistical Classification of Diseases and Related Health Problems, tenth revision diagnostic codes.

© 2022 Räikkönen K et al. *JAMA Network Open.*

eTable 4. Adjusted Hazard Ratios (HR) of Psychological Developmental and Neurosensory Disorders in Children Born in 2006-2008 and 2009-2017 According to Maternal Antenatal Corticosteroid Treatment Exposure

	Children born in 2006-2008 N=170361[a]		Children born in 2009-2017 N=499736[a]	
**Outcome**	**Adjusted HR (95% CI)**	***P***	**Adjusted HR (95% CI)**	***P***
**Psychological developmental disorders**				
Specific developmental disorders of speech and language (F80)	1.56 (1.31 to 1.85)	<.001	1.26 (1.15 to 1.39)	<.001
Specific developmental disorders of scholastic skills (F81)	1.59 (1.27 to 1.99)	<.001	1.05 (0.85 to 1.30)	.64
Specific developmental disorder of motor function (F82)	1.59 (1.26 to 1.99)	<.001	1.19 (1.03 to 1.36)	.01
Pervasive developmental disorder (F84)	1.53 (1.17 to 2.00)	.002	1.19 (1.00 to 1.42)	.05
Other or unspecified disorder of psychological development (F88, F89)	2.34 (1.71 to 3.20)	<.001	1.55 (1.25 to 1.92)	<.001
**Neurosensory disorders**				
Vision or hearing loss (H54, H90, H91)	1.43 (1.10 to 1.86)	.008	1.12 (0.92 to 1.38)	.26
Epilepsy (G40, G41)	0.89 (0.62 to 1.29)	.54	1.13 (0.91 to 1.39)	.27
Cerebral palsy (G80)	1.17 (0.77 to 1.78)	.55	1.18 (0.92 to 1.52)	.22

[a]In adjusted analyses sample size for the cohort of children born in 2006-2008 is 161931, and for the children born in 2009-2017 it is 480227.

F-, G-, and H-codes in parenthesis refer to International Statistical Classification of Diseases and Related Health Problems, tenth revision diagnostic codes.

© 2022 Räikkönen K et al. *JAMA Network Open.*
